# Hyperspectral identification of oil adulteration using machine learning techniques

**DOI:** 10.1016/j.crfs.2024.100773

**Published:** 2024-05-22

**Authors:** Muhammad Aqeel, Ahmad Sohaib, Muhammad Iqbal, Hafeez Ur Rehman, Furqan Rustam

**Affiliations:** a Advance Image Processing Research Lab (AIPRL), Institute of Computer & Software Engineering, Khwaja Fareed University of Engineering and Information Technology, Rahim Yar Khan, 64200, Pakistan; b Center of Artificial Intelligence and Cyber Security, Khwaja Fareed University of Engineering and Information Technology, Rahim Yar Khan, 64200, Pakistan; c Institute of Computer Science, Khwaja Fareed University of Engineering and Information Technology, Rahim Yar Khan, Pakistan; d School of Computer Science, University College Dublin, Dublin, D04V1W8, Ireland

**Keywords:** Adulteration, Edible oil, Food quality control, Hyperspectral imaging, Artificial intelligence, Machine learning

## Abstract

Food adulteration is a global concern, drawing attention from safety authorities due to its potential health risks. Detecting and categorizing oil adulteration is crucial for consumer safety and food industry integrity. This research explores hyperspectral imaging (HSI) analysis to identify substandard oil adulteration at different stages. Using the non-destructive HSI Specim Fx 10 system, a method for precise and easy imaging-based fraud detection and classification was proposed. The 670 oil samples, including pure (Almond, Mustard, Coconut, Olive) and adulterated (Sunflower, Castor, Liquid Paraffin), were analyzed. The Savitzky-Golay filter preprocessed the images to remove noise and smooth spectral signatures. The oils were identified using various machine learning approaches, including Support Vector Machines, Logistic Regression, Linear Discriminant Analysis, Random Forests, Decision Trees, K-Nearest Neighbors, and Naïve Bayes with Linear Discriminant Analysis excelling in identification. Performance parameters, including precision, recall, F1-score, and overall accuracy, were calculated. The proposed method achieved a validation accuracy of 100%, outperforming numerous state-of-the-art approaches. This study introduces a robust pipeline for effective oil adulteration detection, offering a significant advancement in food safety and quality control.

## Highlights

• Employed hyperspectral imaging and machine learning to identify pure and adulterated oils, addressing global food safety.
• Processed various oil samples with a Savitzky-Golay filter; Linear Discriminant Analysis achieved 98% validation accuracy.
• Hyperspectral imaging covered the 400-1000nm spectral range, detecting oil adulteration throughout the supply chain.
• The proposed method classifies pure and adulterated oils offering a solution to combat food fraud and ensure consumer health.

## Graphical abstract

**Figure:**
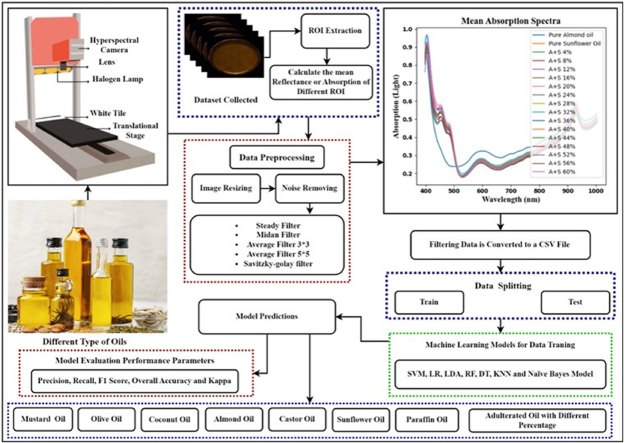

## 1 Introduction

Manual detection and classification of oil adulteration in the food industry are acknowledged as time-consuming, prone to inaccuracies, and challenging (Johnson, 2014). This concern stems from the significant risks to public health and the erosion of consumer trust caused by the pervasive issue of oil adulteration (Chaudhari et al., 2021). The growing global challenge of adulterated cooking oil has adverse health effects, making individuals more susceptible to diseases such as cancer and cardiac conditions. An alarming case in Spain, where rapeseed oil adulteration led to 600 deaths and affected 300 individuals, underscores the severity of this issue (Posada De La Paz et al., 2001). To address these challenges, an intelligent system for automatic oil adulteration detection and classification is imperative (Samuel and Geetha, 2014). Despite the critical role edible oils play in human nutrition (Lima and Block, 2019; Gonzalez-Ortega et al., 2024), challenges in their production and quality control persist (Review, 2023), with oil adulteration detection being a significant hurdle (Wei et al., 2023; Wahrburg et al., 2002). Adulteration involves the addition of cheaper oils or impurities to pure oil to enhance volume, shelf life, and appearance (Pal, 2011). Various detection methods, including physical and chemical tests (Heuscher et al., 2005), gas chromatography (Abed and Khairy, 2023), and high performance liquid chromatography (Abed et al., 2023), have been explored but are often time-consuming, costly (Portarena et al., 2019), and not universally effective. In response to these challenges, hyperspectral imaging (HSI) emerges as a promising solution, combining spectral and spatial information analysis with machine learning (ML) algorithms for rapid and accurate detection of various oil adulterants (Lozano-Garzón et al., 2023; Choudhary et al., 2020).

The proposed intelligent system employs HSI technology to capture spectral and spatial data from oil samples, which is then analyzed using ML algorithms like Linear Regression (LR) (Mohammadinia et al., 2023), Random Forest (RF) (Tie et al., 2024), Decision Tree (DT) (Raj et al., 2023), Support Vector Machine (SVM) (Lu et al., 2023), K-nearest Neighbor (KNN) (Saifullah et al., 2023), and Linear Discriminant Analysis (LDA) (da Cruz Souza et al., 2023). HSI's non-destructive nature and high accuracy offer the potential to detect a wide range of adulterants (Wang et al., 2023). The system aims to revolutionize oil adulteration detection by providing a more comprehensive understanding of the chemical characteristics of oil samples. The study involves four pure oils—Almond, Mustard, Coconut, and Olive—adulterated with Sunflower, Castor, and liquid Paraffin. Machine-learning algorithms, including SVM, LR, LDA, RF, DT, KNN, and Naïve Bayes, are applied for oil adulteration classification, with the ultimate goal of designing an intelligent, fast, accurate, and cost-effective system for the food industry.

The research methodology includes the acquisition of oil samples, HSI data capture using a Specim Fx 10 push broom/line scanner system, determination of reflectance and absorption, and estimation of mean absorption spectra signatures for Regions of Interest (ROI). Pre-processing involves noise removal and smoothing using the Savitzky-Golay filter, followed by conversion into data text or CSV files for further analysis. A novel algorithm for oil adulteration detection and classification using ML models is proposed, with LDA identified as the optimal model. Performance parameters are calculated for model evaluation, highlighting the system's effectiveness in detecting and preventing oil adulteration in the food industry.

In conclusion, the integration of hyperspectral imaging and ML algorithms presents a promising and innovative approach to revolutionize oil adulteration detection and classification in the food industry, ensuring the quality and safety of edible oils. The proposed intelligent system addresses the limitations of traditional methods and offers a comprehensive solution to the complex and global challenge of oil adulteration. This paper makes the following contributions.

The study conducted a thorough analysis of various commercial oils, including paraffin, coconut, almond, mustard, castor, olive, and sunflower oils. Utilized a push broom/line scanner HSI system (Specim Fx 10) to obtain high-resolution spectral data (224 bands) with spatial information for each oil sample. Implemented the Lumo Scanner software for data processing, calculating reflectance, and generating absorption spectra signatures for each oil sample. Employed noise removal techniques such as median, Savitzky-Golay, steady, and moving average filters to enhance the quality of the spectral data. Trained machine learning models (SVM, LR, LDA, RF, DT, KNN, and Naïve Bayes) using preprocessed spectral data to distinguish between pure and adulterated oil classes. Computed various performance metrics, including recall, kappa, F1-score, overall accuracy, and precision, to assess the reliability and efficiency of the models.

## 2 Materials and methods

Although adulteration in edible oil is a major problem that negatively impacts human health worldwide, edible oil plays a vital role in preserving human health. The adulteration of expensive and mostly useable cooking oil in Pakistan is the subject of this study. The following procedures have been used in conducting the research.

**Fig. 1:**
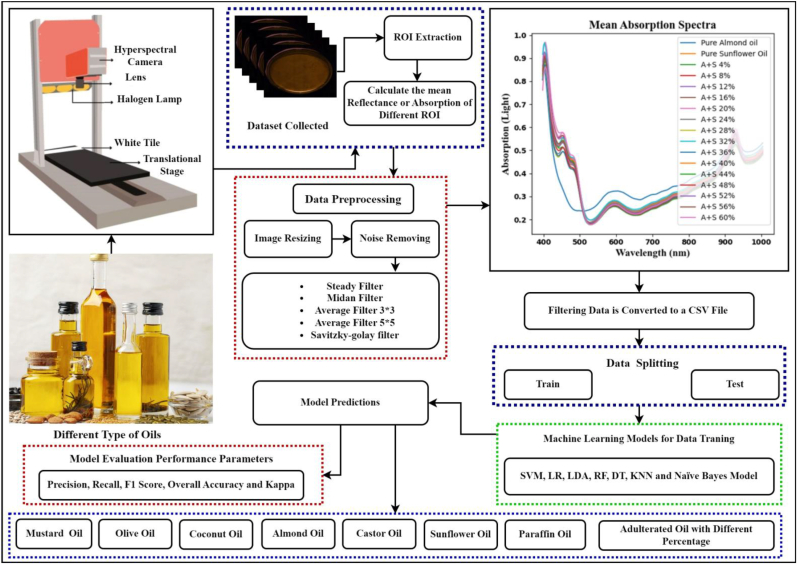
HSI based proposed model.

• Samples of paraffin, coconut, almond, mustard, castor, olive, and sunflower oils were bought from the commercial market. Pure Almond, Coconut, Olive, and Mustard oils are tainted with Sunflower, Liquid Paraffin, and Castor oils. In the second phase, oil samples were obtained utilizing a push broom/line scanner HSI system (Specim Fx 10). Using Lumo Scanner software, 224 spectral bands (λ) with 100 × 512 spatial pixels were attained for the acquired sample.
• Reflectance was calculated using the oil data that was collected. This work allows the use of a complete hyperspectral imaging (HSI), however large datasets are required for machine learning model training. As such, we choose to extract several Regions of Interest (ROIs) from a single image. Selected pixel values are used to create these ROIs, which contain spatial information. Since every pixel contains a spectrum of spectral data, we calculate the average of these spectra. For every ROI that was chosen, the mean reflectance spectra signature was determined. But because oil has the ability to absorb light, the reflectance spectra were used to calculate the absorption spectra signature for each oil sample. From each mean ROI, 224 features in total were extracted. In order to facilitate smooth preprocessing, noise and other undesired effects were removed from the spectral signature of the raw, captured oil samples (pure and tampered with). The noise in the oil samples was removed using median, Savitzky-Golay, steady, and moving average filters; the spectral signature was smoothed using smoothing filters. The Savitzky-Golay filter was found to be the most effective among other filters at removing noise and smoothing the samples' spectral signatures after a comparison of all the resulting spectral signatures.
• The entire dataset was divided into training (80%) and testing (20%) files after the Savitzky-Golay spectral signatures were converted to data text or a CSV file. ML models were trained using preprocessed spectral data. The model is a computer programme that is capable of learning to distinguish between various pure and adulterated classes based on specific spectral signatures. The pure and adulterated classes were predicted by the ML models (SVM, LR, LDA, RF, DT, KNN, and Naïve Bayes) using a spectral signature.
• In addition, it has been determined that LDA is the most effective model that has been suggested for the detection and classification of oil adulteration after comparing these ML models. Ultimately, performance metrics such as recall, kappa, F1-score, overall accuracy, and precision were computed to assess the model. The suggested model's general workflow is shown in Fig. 1.

### 2.1 Dataset collection and acquisition

An HSI camera (FX-10, Specim, Spectral Imaging Ltd., and Oulu, Finland) was used in this experiment. The camera came with a unique Scheiner lens (Cinegon 1.4/8 mm) preinstalled. The camera was placed on a lab scanner to scan the entire sample because it operates on the line scan principle, which involves scanning one thin line of the object with its complete spectrum at a time. The scanner is equipped with three movable 40 × 20 cm platforms, a mounting plate for the camera, and three halogen lights for illumination. The camera mounting plate can be adjusted in height so that it always sits 6 cm above the sample. After a few cycles, the height was adjusted to ensure that the field of vision closely matched the sample. The camera's spatial resolution was enhanced.

Samples of mustard, castor, sunflower, almond, olive, coconut, and paraffin oil were bought from a local commercial market in Pakistan. In order to represent the contaminated oil, which ranges in volume from 1 to 25 ml, 10 samples of each pure oils were first taken. During the adulteration process, varying concentrations of different oils were added in increments from 1 ml to 15 ml, with 10 samples taken for each concentration. The all oils were treated in the same way: pure almond oil was tainted with sunflower oil; pure mustard oil was tainted with castor oil; pure olive oil was tainted with sunflower oil and last, pure coconut oil was used as pure oil and tainted with liquid paraffin. We were able to produce 70 pure samples and 600 contaminated samples using this method. Table .1 contains comprehensive details about every oil sample. Purified castor oil (class “1″), pure mustard oil (class “2″), pure olive oil (class “3″), pure sunflower oil (class “4″), pure coconut oil (class “5″), pure liquid Paraffin oil (class “6″), pure almond oil (class “7″), pure mustard + castor oil adulterated (class “8″), pure olive + sunflower oil adulterated (class “9″), pure coconut + liquid Paraffin oil adulterated (class “10″), and pure almond + sunflower oil adulterated (class “11”). There are 5500 rows in the CSV file, with 224 columns.

**Table 1:** Samples prepared for each pure and adulterant.

Samples Details	No of Samples
Pure Castor Oil 25 ml	10
Pure Mustard Oil	10
Pure Mustard + Castor Oil Adulterated (1 ml–15 ml)	150
Pure Olive Oil 25 ml	10
Pure Sunflower Oil 25 ml	10
Pure Olive + Sunflower Oil Adulterated (1 ml–15)	150
Pure Coconut Oil 25 ml	10
Pure Liquid Paraffin Oil 20 ml	10
Pure Coconut + Liquid Paraffin Oil Adulterated (1 ml–15 ml)	150
Pure Almond Oil 25 ml	10
Pure Almond + Sunflower Oil Adulterated (1 ml–15 ml)	150
**Total**	**670**

Each row in the dataset represents a specific observation by each selected ROI, which could correspond to an HSI captured from a particular oil sample. The 224 columns may correspond to different spectral bands or features extracted from each pixel within the hyperspectral recorded image. These spectral bands capture information across various wavelengths, allowing for a detailed characterization of the chemical composition of the oil. The dataset contains spectral data for both pure and adulterated oil samples. Each row would represent a different selected ROI spectral information of the oil sample, while the columns would represent the spectral features extracted from the hyperspectral image of that sample.

**Fig. 2:**
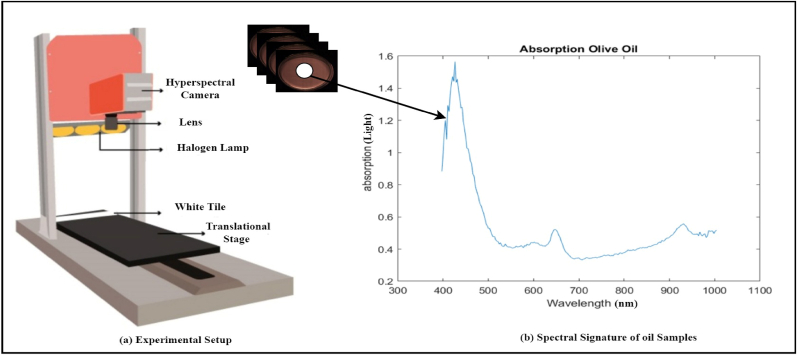
HSI experimental setup and spectral signature of the oil.

**Fig. 3:**
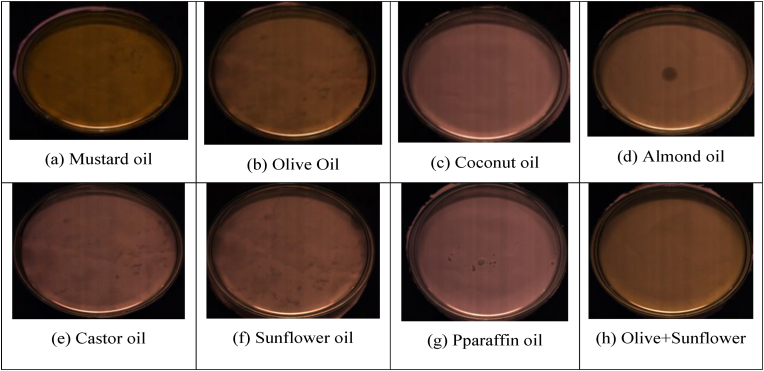
Visual difference of the pure oil classes (a–g) and adulterated oil (h).

• A Petri dish with the prepared samples in it is seen in Fig. 2. Samples were levelled using a surface leveler to avoid shadowing and blurring. The levelled samples were put on the scanner's moving bed. As a point of comparison, a white tile with a 99.9% reflectance rating was set up on the moving platform next to the samples. The reference item was placed in the same spot as each sample for the purpose of calculating reflectance. Next, the platform was configured to move at a speed of 20 m/s, and the frame rate of the camera was set to 50 Hz. The aperture gathering samples of the lens was manually adjusted for each example in order to adjust the exposure. The sample data was gathered using the methodology described below (see Fig. 3).
  • With the camera's shutter closed, 100 dark frames were captured. Many frames were recorded in order to capture the sensor's response in its entirety.
  • The camera captured 100 frames of the white tile, with the white reference placed where the lab scanner translational platform had been relocated.
  • The translational platform was moved to the sample's location and line-by-line scanned.

The Lumo Scanner Software was updated to alter the white reference, target start, and target stop locations in addition to the ratio of dark to white frames in order to control the position of the global platform and sample collection. Three raw files in total—a dark reference, a white reference, and the sample—were retained for every sample. Using the object's spectral responses at 430, 510, and 670 nm, a false-color RGB image of the scanned sample was produced in addition to these files. The sample and reference data were loaded into MATLAB as a 3D cube using the multiband-read command.

### 2.2 Spectral reflectance

The spectral radiance that the hyperspectral sensor records is influenced by various factors such as atmospheric effects, illumination, object geometry, and sensor properties, which can lead to inaccurate results. To account for those effects, the imperial line approach was used to determine the object's true reflectance. In order to eliminate atmospheric effects from reflectance calculations, a dark and white reference is required. An opaque black cap was used to completely cover the camera lens in order to obtain the dark reference B, and a white tile was scanned in order to record the white reference W. All of the samples were prepared in accordance with equation (1).

(1) $$R=\frac{\text{Rad}-B}{W-B}$$

Where Rad is the spectral radiance recorded by the HSI-system, with 0% reflectance for B and 99.9% for W, and R is the normalized accurate reflectance cube. Additionally, by utilizing the oil reflectance, it is able to compute each oil absorption spectral signature because of its transparency. Equation (2) provides a formula for computing the absorption values of oil samples.

(2) $$A=\mathrm{Log}\;\left(\frac{1}{R}\right)$$

where A stands for absorption and R for the cube values of reflectance. The chemical bond composition is represented by the computed absorption spectrum.

### 2.3 Classification methods

Hyperspectral imaging offers a sophisticated approach to detect oil adulteration by analyzing spectral signatures across a broad range of wavelengths. This method leverages the unique spectral properties of different oil types, allowing for precise classification and identification of adulterants. By capturing a multitude of spectral bands, hyperspectral imaging provides detailed information about the chemical composition of oils, enabling the detection of even subtle alterations. Advanced classification algorithms (SVM, LR, LDA, RF, DT, KNN, and Naïve Bayes) are employed to interpret the spectral data and distinguish between genuine oils and adulterants, facilitating rapid and accurate detection. This technology holds significant promise for ensuring the authenticity and quality of oils in various industries, including food, pharmaceuticals, and cosmetics, ultimately safeguarding consumer health and trust.

#### 2.3.1 Support Vector Machine

SVM can be used in the context of HSI data-based oil adulteration detection and classification to precisely distinguish between pure and adulterated oil. The best hyperplane in a high-dimensional feature space for separating data points into different classes is found by the SVM model. In this work, pure and diluted oils at varying concentrations make up the classes, and the spectral signatures taken from the HSI data define the feature space. Preprocessed data are separated into two sets: testing and training. The model is trained on the training set, and its performance is assessed using a 10-fold test on the testing set for cross validation. The typical metrics are used to assess the model's performance in accurately classifying pure and adulterated oils at various stages, including accuracy, precision, recall, F1-score, and kappa. Optimizing the hyperparameters kernel = 'linear’, C = 10, decision_function_shape = ‘ovo’, and random_state = 10 is necessary to attain optimal performance in SVM.

#### 2.3.2 Logistic Regression

A potent ML algorithm called LR Model for HSI Data was created to correlate target variables with spectral information that was taken from hyperspectral images. Because the features and the target have a linear relationship, it is perfect for qualitative analysis and estimation of pure and adulterated oils at various stages. To predict the target adulterated and pure class for newly discovered, unseen hyperspectral images, the LR model is trained on labelled hyperspectral data. Table .2 shows how 10-fold metrics like recall, accuracy, precision, F1-score, and kappa value are used to assess the performance of the model.

**Table 2:** Proposed model evaluation performance parameter comparisons.

List of Models	No of Classes	Precision	Recall	F1-Score	Kappa	Overall Accuracy
Smoothing + SVM	Pure Castor Oil	100%	100%	100%	100%	100%
	Pure Mustard Oil	100%	100%	100%	100%	100%
	Pure Olive Oil	100%	100%	100%	100%	100%
	Pure Sunflower Oil	100%	100%	100%	100%	100%
	Pure Coconut Oil	100%	100%	100%	100%	100%
	Pure Liquid Parafin Oil	100%	100%	100%	100%	100%
	Pure Almond Oil	100%	100%	100%	100%	100%
	Pure Mustard + Castor Oil Adulterated	100%	100%	100%	100%	100%
	Pure Olive + Sunflower Oil Adulterated	100%	100%	100%	100%	100%
	Pure Coconut + Liquid Parafin Oil Adulterated	100%	100%	100%	100%	100%
	Pure Almond + Sunflower Oil Adulterated	100%	73%	85%	83%	97%
Smoothing + LR	Pure Castor Oil	100%	100%	100%	100%	100%
	Pure Mustard Oil	79%	88%	83%	81%	96%
	Pure Olive Oil	100%	100%	100%	100%	100%
	Pure Sunflower Oil	100%	100%	100%	100%	100%
	Pure Coconut Oil	100%	100%	100%	100%	100%
	Pure Liquid Parafin Oil	100%	100%	100%	100%	100%
	Pure Almond Oil	95%	98%	97%	96%	99%
	Pure Mustard + Castor Oil Adulterated	87%	78%	82%	80%	96%
	Pure Olive + Sunflower Oil Adulterated	100%	100%	100%	100%	100%
	Pure Coconut + Liquid Parafin Oil Adulterated	100%	100%	100%	100%	100%
	Pure Almond + Sunflower Oil Adulterated	97%	95%	96%	96%	99%
Smoothing + LDA	Pure Castor Oil	100%	100%	100%	100%	100%
	Pure Mustard Oil	100%	100%	100%	100%	100%
	Pure Olive Oil	100%	100%	100%	100%	100%
	Pure Sunflower Oil	100%	100%	100%	100%	100%
	Pure Coconut Oil	100%	100%	100%	100%	100%
	Pure Liquid Parafin Oil	100%	100%	100%	100%	100%
	Pure Almond Oil	100%	100%	100%	100%	100%
	Pure Mustard + Castor Oil Adulterated	100%	100%	100%	100%	100%
	Pure Olive + Sunflower Oil Adulterated	100%	100%	100%	100%	100%
	Pure Coconut + Liquid Parafin Oil Adulterated	100%	100%	100%	100%	100%
	Pure Almond + Sunflower Oil Adulterated	100%	100%	100%	100%	100%
Smoothing + RF	Pure Castor Oil	100%	100%	100%	100%	100%
	Pure Mustard Oil	85%	98%	91%	90%	98%
	Pure Olive Oil	100%	100%	100%	100%	100%
	Pure Sunflower Oil	99%	100%	99%	99%	99%
	Pure Coconut Oil	100%	99%	99%	99%	99%
	Pure Liquid Parafin Oil	100%	100%	100%	100%	100%
	Pure Almond Oil	100%	100%	100%	100%	100%
	Pure Mustard + Castor Oil Adulterated	97%	84%	90%	89%	98%
	Pure Olive + Sunflower Oil Adulterated	100%	100%	100%	100%	100%
	Pure Coconut + Liquid Parafin Oil Adulterated	100%	100%	100%	100%	100%
	Pure Almond + Sunflower Oil Adulterated	100%	100%	100%	100%	100%
Smoothing + DT	Pure Castor Oil	100%	100%	100%	100%	100%
	Pure Mustard Oil	72%	94%	81%	79%	96%
	Pure Olive Oil	100%	100%	100%	100%	100%
	Pure Sunflower Oil	94%	98%	96%	95%	99%
	Pure Coconut Oil	98%	97%	97%	97%	99%
	Pure Liquid Parafin Oil	100%	100%	100%	100%	100%
	Pure Almond Oil	100%	100%	100%	100%	100%
	Pure Mustard + Castor Oil Adulterated	91%	64%	75%	73%	96%
	Pure Olive + Sunflower Oil Adulterated	100%	100%	100%	100%	100%
	Pure Coconut + Liquid Parafin Oil Adulterated	100%	100%	100%	100%	100%
	Pure Almond + Sunflower Oil Adulterated	97%	96%	96%	96%	99%
Smoothing + KNN	Pure Castor Oil	100%	100%	100%	100%	100%
	Pure Mustard Oil	69%	82%	75%	72%	94%
	Pure Olive Oil	100%	100%	100%	100%	100%
	Pure Sunflower Oil	94%	97%	96%	95%	99%
	Pure Coconut Oil	72%	91%	80%	78%	96%
	Pure Liquid Parafin Oil	100%	100%	100%	100%	100%
	Pure Almond Oil	100%	100%	100%	100%	100%
	Pure Mustard + Castor Oil Adulterated	75%	59%	66%	63%	94%
	Pure Olive + Sunflower Oil Adulterated	100%	100%	100%	100%	100%
	Pure Coconut + Liquid Parafin Oil Adulterated	100%	100%	100%	100%	100%
	Pure Almond + Sunflower Oil Adulterated	95%	69%	80%	78%	96%
Smoothing + Naïve Bayes	Pure Castor Oil	100%	93%	96%	96%	99%
	Pure Mustard Oil	77%	86%	81%	79%	96%
	Pure Olive Oil	89%	100%	94%	93%	98%
	Pure Sunflower Oil	17%	7%	10%	6%	89%
	Pure Coconut Oil	55%	66%	60%	55%	91%
	Pure Liquid Parafin Oil	93%	92%	92%	92%	98%
	Pure Almond Oil	65%	95%	77%	74%	94%
	Pure Mustard + Castor Oil Adulterated	85%	76%	80%	78%	96%
	Pure Olive + Sunflower Oil Adulterated	100%	86%	92%	91%	98%
	Pure Coconut + Liquid Parafin Oil Adulterated	100%	100%	100%	100%	100%
	Pure Almond + Sunflower Oil Adulterated	45%	36%	40%	35%	90%

#### 2.3.3 Linear Discriminant Analysis

Oil adulteration in hyperspectral imaging data is detected and classified using LDA, a supervised ML technique. To maximize the separation between various classes of oil samples, such as pure oil and adulterated oil, a linear combination of spectral bands is used. Following hyperspectral data preprocessing and the extraction of pertinent spectral signatures from ROI, LDA is applied. It assists in finding trends in the data that set pure oil samples apart from tainted ones. Table .2 illustrates how feature selection, appropriate model evaluation, and data quality all affect how effective LDA is.

#### 2.3.4 Random Forest

In order to identify and categorize oil adulteration in HSI data, RF is a potent technique. To create precise classifications, it builds several decision trees during training and merges their forecasts. High-dimensional data is a good fit for RF, which can also automatically choose key spectral characteristics to differentiate between pure and tainted oils samples. It can handle noisy data, is resilient, and is less likely to overfit. RF is a useful tool for tasks involving the detection and classification of oil adulteration because of its capacity to provide feature importance rankings, which aid in the understanding of the key discriminative features in the data. A summary of each class's classification is shown in Table .2.

#### 2.3.5 Decision Tree

The DT is used to identify and categorize oil adulteration in HSI data. It divides the data recursively according to various spectral features, resulting in a structure resembling a tree. Every leaf node represents the class (pure or adulterated oil), and every internal node represents a choice made in response to a particular feature. The use of multiple DTs combined to increase classification accuracy and robustness.

#### 2.3.6 K-Nearest Neighbors

KNN works by using a similarity metric, like Euclidean distance, to find the two-nearest data points (samples) to a new data point (test sample). The majority of the k-nearest neighbors are used to categorize samples of pure and contaminated oil. KNN is simple to use and understand because it is non-parametric and doesn't require any training when creating the model. For the detection and classification of oil adulteration, KNN is a helpful model, particularly in cases where the dataset is small. The classification report for each class is displayed in Table .2.

#### 2.3.7 Naïve Bayes

In HSI data, oil adulteration is detected and classified using the Naïve Bayes model. The method, which is based on the Bayes theorem, determines which class a sample belongs to base on the observed features and computes the probability that the sample belongs to a pure and adulterated class. It functions effectively with high-dimensional data and is computationally efficient. The classification summary reports for every class are displayed in Table .2.

## 3 Results and discussion

This study presents the most inventive and dependable approach to oil adulteration detection and classification using hyperspectral imaging and ML. The experimental results show that the various ML techniques combined in this study provide an accurate, sensitive, and effective way to guarantee the quality and authenticity of edible oils. Improved consumer safety and increased trust among various stakeholders are two potential advantages of the suggested model for the food industry, which rely on its importance in preserving the integrity of the global food supply chain. To preserve human health and reduce health risks that could ultimately endanger lives, we proposed a method to detect adulteration in cooking oils. By gathering an enormous amount of spectral information for every pixel in an image over a broad range of wavelengths, HSI provides a state-of-the-art solution. A thorough understanding of the physical and chemical makeup of the substances being investigated is possible thanks to this high-dimensional data. HSI can be used to distinguish between genuine oils and those that have been tainted or combined with inferior oils when analyzing edible oils. Furthermore, the suggested hyperspectral (400–1000) imaging-based model's non-destructive and instantaneous checking properties make it a perfect tool for quality assurance and control in the food processing sector. The model provides both spatial and spectral data for pure and contaminated oil. Certain costly and widely used cooking oils are utilized in developing countries like Pakistan. Samples of locally accessible liquid paraffin oil, castor, sunflower, and coconut oils, as well as pure mustard, almond, olive, and coconut oils and their adulterants, were used in this investigation. Following that, spectral signature was computed using each oil's reflectance to determine the absorption spectral signature for each oil sample due to oil transparency. The suggested Savitzky-Golay filter increases the accuracy of the built model by eliminating noise from the spectral signature. The Naïve Bayes, SVM, LR, LDA, RF, DT, and KNN models are used for classification.

### 3.1 Preprocessing of spectral analysis

**Fig. 4:**
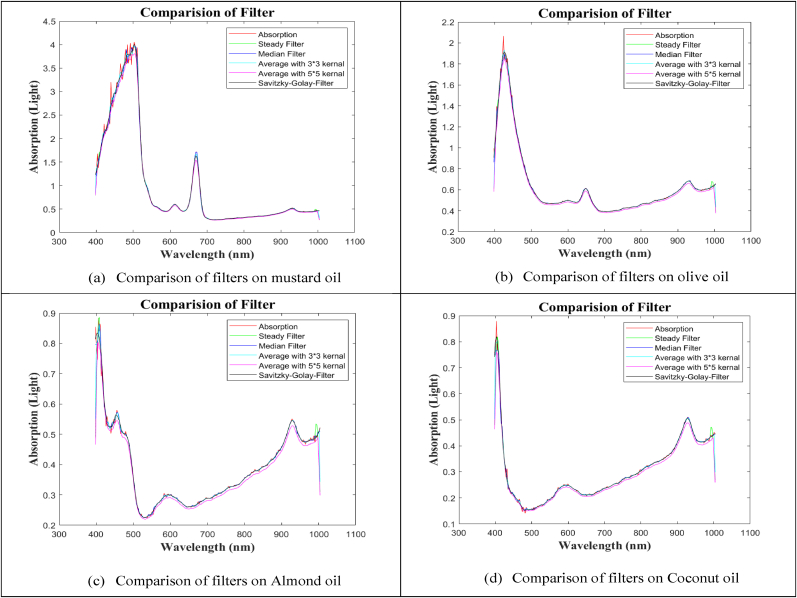
Compressed spectral signatures of oils (a–d) using absorption, steady, median, average, and Savitzky-Golay smoothing filters.

Although the spectral signature of oil sample absorption contains extremely sensitive data, it also contains systematic noise (scattering and ambient light). These noises have the potential to introduce errors into the obtained spectral data and compromise the ML model's accuracy. Numerous mathematical methods are available to eliminate the noise from the obtained spectral data. Certain mathematical strategies or approaches that work best eliminate the impact of errors from spectral data based on the hyper-cube's nature. In order to remove noise from the obtained spectral data, we employed the following filters: moving average, median, Savitzky-Golay, and steady. We then compared the outcomes to determine which filter performed best. Fig. 4 illustrates the results of comparing all the resulting spectral signatures and finds that the Savitzky-Golay filter works best for removing noise and smoothing the spectral signature of oil samples.

### 3.2 Spectral analysis of pure and adulterated oil samples

Using the spectral characteristics of various oils, spectral analysis offers a quick and non-destructive way to identify oil adulteration. In order to identify adulterants, ensure the integrity and purity of oil products, and monitor adulteration, it can be especially helpful in quality control and monitoring. Through the use of quantity level changes, the spectral characteristics of various adulterants are examined in order to identify distinct signatures that allow for their differentiation.

**Fig. 5:**
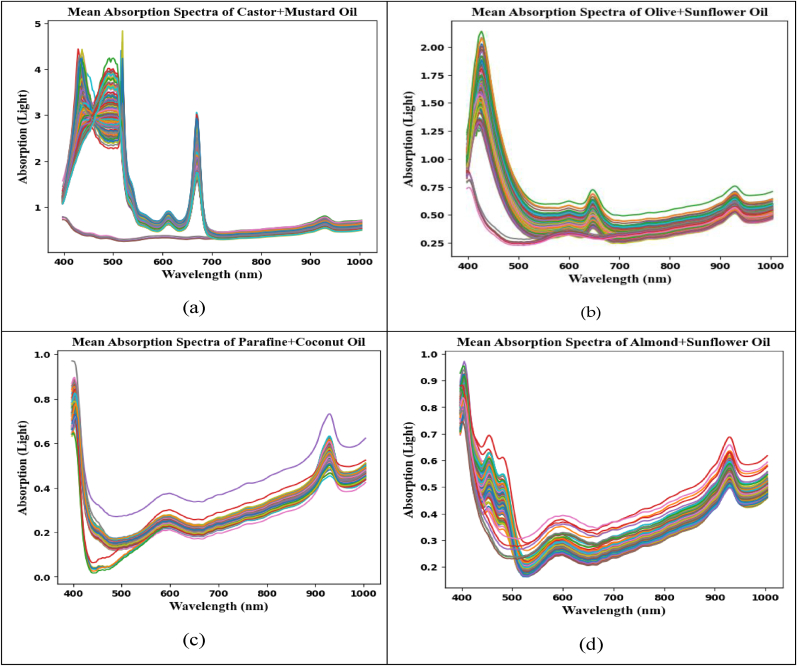
The visualization (a to d) of pixel level HSI spectral signature of different oil samples.

This process is known as spectral analysis. Every pixel in an image has its spectral information recorded by HSI over a wavelength range of (400–1000 nm). Fig. 5 displays the signatures of pure and contaminated oil derived from an analysis of the distinct spectral signatures of various oils.

**Fig. 6:**
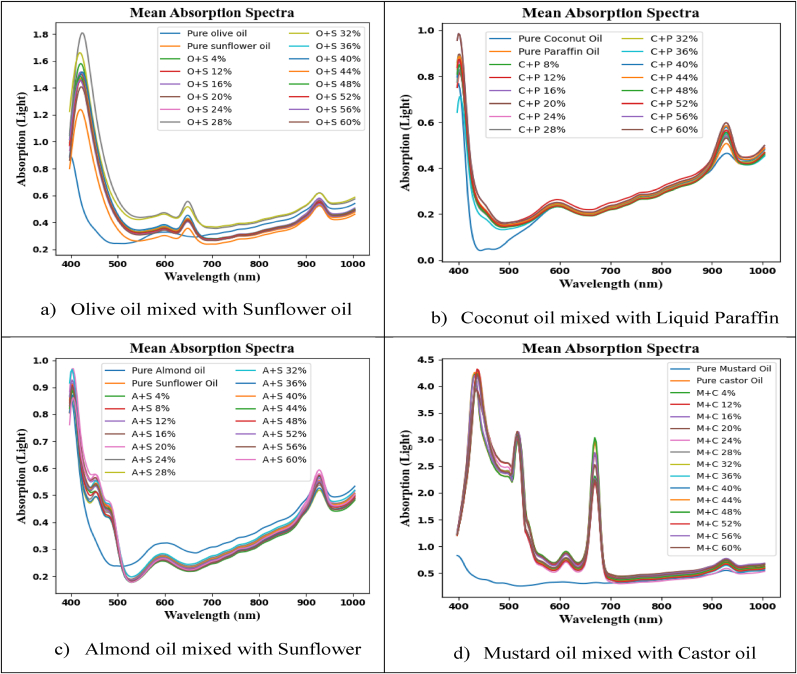
The HSI spectral signature distinguishes pure and adulterated oils (0–60%). (a): Olive and sunflower oils, (b): Coconut and Paraffin oils, (c): Sunflower oil with Almond oil, (d): Castor oil (4–60%) with Mustard oil.

In Fig. 6, the nutritional makeup of contaminated olive and sunflower oils can be compromised. The nutrients found in sunflower and olive oils can vary depending on the number of antioxidants, vitamins, and fatty acids they contain. Buying oils from reliable sources is essential to ensuring their authenticity and quality, as adulteration is both illegal and unethical. Examining the spectral properties of pure and adulterated oil samples allows for the identification of differences and the detection of adulterants. Fig. 6 shows that the majority of the samples' spectral signatures overlap at various spectral line locations. As seen in Fig. 6 (a to d), spectra clearly illustrate all similarities and changes. Each spectral signature displays a sharp curve with varying wavelengths (in nanometers).

## 4 Pipeline models comparison

With HSI, a broad range of electromagnetic wavelengths are captured, resulting in full spectral signature information for every pixel in an image of an oil sample. This allows the spectral signatures of pure and adulterated oils to be distinguished. The objective of oil adulteration detection is to locate and measure any adulterant oil substances present in pure oil. The chemical compositions and spectral signatures of various oils differ. ML models are able to distinguish between the spectral signatures of pure and contaminated oil. ML-based HSI systems identify various degrees of adulteration to guarantee the authenticity and quality of oils. The evaluation parameters for pure and adulterated stage classification are precision, Recall, F1-score, kappa, and overall accuracy. Table 2 illustrates a self-comparison among the recommended pipeline models, offering valuable insights into the strengths and weaknesses inherent in each model. These proposed models undergo training and evaluation across various oil categories, encompassing both pure oils and adulterated oils. The performance metrics of each model are assessed through a comprehensive class classification summary report, including precision, recall, accuracy, F1-score, Kappa, and overall accuracy. The reported percentages extend to 100%, covering eleven distinct classes in this table.

The ± standard deviation reflects variability in accuracy observed among folds. A smaller standard deviation suggests less variability, indicating more consistent performance across different folds, whereas a larger standard deviation indicates greater variability. It helps to assess how well a model generalizes to unseen data by training and testing the model on multiple subsets of the dataset. The newly acquired samples have undergone assessment for external testing, Table 3 presents the performance metrics of the suggested models.

(3) $$\text{Predicted}\;\text{class}\;\text{confidence}=\frac{\mathrm{Max}\;decision\;score-second\;\mathrm{Max}\;decision\;score}{\mathrm{Max}\;decision\;score}$$

**Table 3:** **P**roposed models confidence prediction for unseen data.

Test	Actual Class	Predicted Class	Predicted class confidence of proposed model						
			LR	SVM	DT	RF	LDA	KNN	Naïve Bayes
Test-1	7	7	100%	90%	100%	97%	100%	100%	100%
Test-2	1	1	98%	100%	100%	100%	100%	100%	100%
Test-3	2	2	97%	100%	100%	87%	100%	100%	99%
Test-4	6	6	99%	100%	100%	99%	100%	100%	100%
Test-5	3	3	99%	100%	100%	100%	100%	100%	100%
Test-6	4	4	96%	100%	100%	79%	100%	100%	99%
Test-7	10	10	99%	100%	100%	100%	100%	100%	100%
Test-8	8	8	95%	98%	100%	52%	100%	80%	98%
Test-9	11	11	99%	100%	100%	99%	100%	100%	99%
Test-10	9	9	99%	100%	100%	100%	100%	100%	100%
Test-11	5	5	97%	100%	100%	100%	100%	100%	100%

Several statistical tests have been conducted (overall accuracy, precision, recall, F1-score, and Kappa) for experimental evaluation. The assessment metrics are derived through the subsequent mathematical expressions.

(4) $$OA=\frac{1}{C}\sum_{i=1}^{C}{TP}_{i}$$

(5) $$\text{Precision}=\frac{TP}{TP+FP}$$

(6) $$\text{Recall}=\frac{TP}{TP+FN}$$

(7) $$F1-\text{score}=2\times \frac{Precision\times Recall}{Precision+Recall}$$

(8) $$P_{o}=\frac{TP+TN}{TP+TN+FP+FN}$$

(9) $$P_{e}=P_{Y}+P_{N}$$

(10) PY=TP+FNTP+TN+FP+FN×TP+FNTP+TN+FP+PN

(11) $$P_{N}=\frac{TN+Fp}{TP+TN+FP+FN}\times \frac{TN+FN}{TP+TN+FP+PN}$$

where FP and TP are false positive and true positive respectively, and FN and TN are false negative and true negative respectively, computed from the performance matrix.

(12) $$\text{Kappa}=\frac{P_{o}-P_{e}}{1-P_{e}}$$

(13) $$K-\text{Fold}\;\text{cross}\;\text{validation}\;\text{accuracy}=\frac{OA+Precision+Recall+F1-score+kappa}{5}$$

(14) $$\text{Standard}\;\text{Deviation}\;\left(\text{SD}\right)=\sqrt{\sum_{i=1}^{k}{\left(x_{i}-\bar{x}\right)}^{2}}$$

$x_{i}$ is the performance score for fold *i,* $\bar{x}$ is the mean performance score across all folds, k is the number of folds in the cross-validation. The stability of the model's performance over the course of the ten folds and the corresponding training time are also shown in the table. Out of all the tested samples, 100% of the samples are correctly classified in the accuracy column. The precision and recall columns show that 100% of all tested oil samples have true positive predictions. The F1-Score, which balances the two metrics by calculating the harmonic mean of recall and precision, has a range of up to 100%. After comparison, LDA is found to be the first most time-efficient and most accurate model. With eleven classes considered, the average accuracy ranges for each model are 95%–100% overall, and the ranges for precision, recall, F1-score, and kappa are (77%–100%, 76%–100%, 75%–100%, and 73%–100%) respectively.

Table .4 provides a thorough post-spectral data processing comparison of the performance demonstrated by various research approaches (SVM, LR, LDA, RF, DT, KNN, and Naïve Bayes) used in the published study. The evaluation uses a 10-fold cross-validation method and includes multiple classification metrics such as recall, precision, F1-score, kappa, and overall accuracy. Utilizing 10-fold cross-validation to assess the robustness and generalization ability of the classifiers. Cross-validation involves splitting the dataset into 10 subsets, training the model on different subsets, and evaluating its performance on the remaining data (see Table 5).

**Table 4:** Self-comparison of the proposed pipeline ML models.

Sr. No	Methodology	Accuracy	Precision	Recall	F1-Score	Kappa	K-Fold ± Standard deviation	Time (s)
1	Smoothing + SVM	100%	100%	98%	99%	98%	0.969 ± 0.0077	0.9693
2	Smoothing + LR	99%	96%	96%	96%	96%	0.947 ± 0.0014	0.9995
3	Smoothing + LDA	100%	100%	100%	100%	100%	1.000 ± 0.0000	1.0000
4	Smoothing + RF	99%	98%	98%	98%	98%	0.998 ± 0.0014	0.9982
5	Smoothing + DT	99%	96%	95%	95%	95%	0.985 ± 0.0052	0.9841
6	Smoothing + KNN	98%	91%	91%	91%	90%	0.986 ± 0.0062	0.9859
7	Smoothing + Naïve Bayes	95%	77%	76%	75%	73%	0.782 ± 0.0359	0.7817

**Table 5:** Comparison of the proposed and reported oil adulteration identification techniques.

Pure Oil	Adulterated oil	Method	Accuracy	Technology	Ref.
Camellia oil	Vegetable oils	PARAFAC-LDA, PARAFAC-PLS-DA,	90.6 and 96.7	–	(Wang et al. (2019))
VOOs	Refined foreign oils	NIR Spectroscopy and Traditional Analytical Parameters	89.3	NIR− Spectroscopy	(Gertz et al. (2020))
Honey	Sugar	SVM, KNN etc.	95%	HSI (400–1000 nm)	(Phillips and Abdulla (2023))
Fresh cheese	Starch content	Correlation coefficient (R2)	0.83%	HIS (200–1000 nm)	(Barreto et al. (2018))
Vegetable oil	Low-cost oil	KNN, RF, SVM, PLS, CNN	89, 90, 95%	Fluorescence spectroscopy	(Wu et al. (2023))
Njangsa seed oil, palm kernel oil, coconut oil, njangsa seed oil-palm kernel oil and njangsa seed oil-coconut oil	Sunflower oil and canola-flaxseed oil margarine	LR, SVM, DT	93,83, 50%	ATR-FTIR Spectroscopy	(Tachie et al. (2024))
Camellia oil (CAO)	Vegetable oil	CNN	95,92	Fluorescence fingerprint	(Chen et al. (2023))
High quality engine oils	Lower quality	PLSR, PCA	90%	Visible/Near Infra-Red Spectroscopy (Vis/NIRS)	(Srata et al. (2019))
Vegetable oil	Palm olein, soybean oil and canola oil	PCA, PLS, KNN, LDA	99%	Portable NIR spectrometer	(Kaufmann et al. (2022))
Camellia oil	Corn oil, rapeseed oil, and sunflower oil	LDA, PLS	99%	Near-infrared spectroscopy and chemometrics	(Du et al. (2021))
Sacha Inchi (SI) oil	vegetable oils	OC-PLS, and DD-SIMCA	89, 93%	NIR spectrometer	(Cruz-Tirado et al. (2023))
Palm oil	–	SIMCA and PLS	99%	Portable NIR spectroscopy	(Basri et al. (2017))
Almond, Mustard, Coconut, and Olive oils	Sunflower, Castor, and Liquid Paraffin	LDA	100%	Hyperspectral Imaging	**This work**

The dataset is divided into ten folds of the equal size. The model is then trained repeatedly ten times, using one fold for validation and nine folds for training in each iteration. This methodical methodology ensures that every data point is used exactly once for validation. This is followed by the averaging of performance metrics over all 10 folds, including kappa, accuracy, precision, recall, F1-score, to approximate the model's generalization performance. This comprehensive analysis provides a broad assessment of the model's effectiveness at every level. This process helps to mitigate the risk of overfitting and provides more reliable estimates of performance. K-Fold cross-validation is a widely used technique for evaluating the performance of ML models. The standard deviation can be used to determine how widely the performance metric is distributed among the k folds and how much the metric deviates from the K-Fold mean performance.

**Fig. 7:**
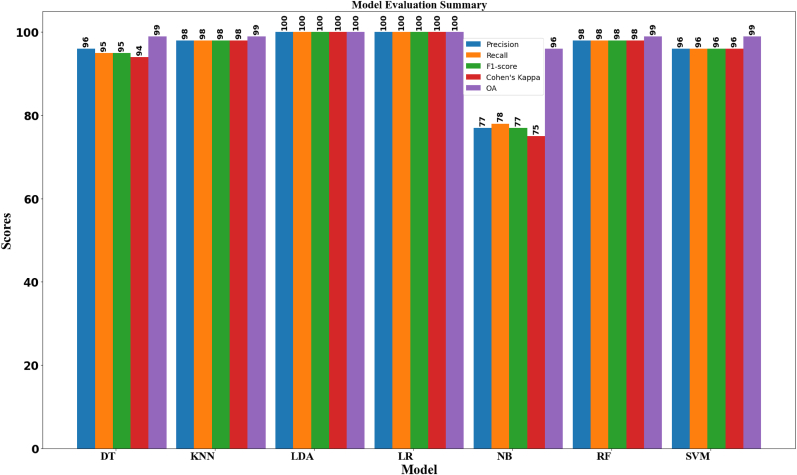
Comparison of performance evaluations of each model.

Fig. 7 summarizes the performance metrics of various applied ML models on different classes spectral signature. The SVM consistently achieves perfect scores across all classes, indicating flawless performance. LR demonstrates high performance overall, with slightly lower scores in some classes compared to SVM. LDA exhibits perfect scores across all classes, showcasing robust performance. RF achieves high scores overall, with some variation in performance across different classes. DT maintains strong performance, albeit with slightly lower scores in certain classes. KNN shows high performance, with some variability across different classes. Naïve Bayes achieves varying levels of performance across each class, with lower scores observed in certain cases. Overall, it demonstrates LDA is decent performance as compared to the other models, indicating their robustness in classifying the pure and adulterated oil data. Evaluation summaries provide valuable information for decision-making processes related to the deployment of classification models in oil adulteration applications. Stakeholders can use these summaries to assess the risks and benefits associated with using a particular model for oil adulteration detection and classification.

**Fig. 8:**
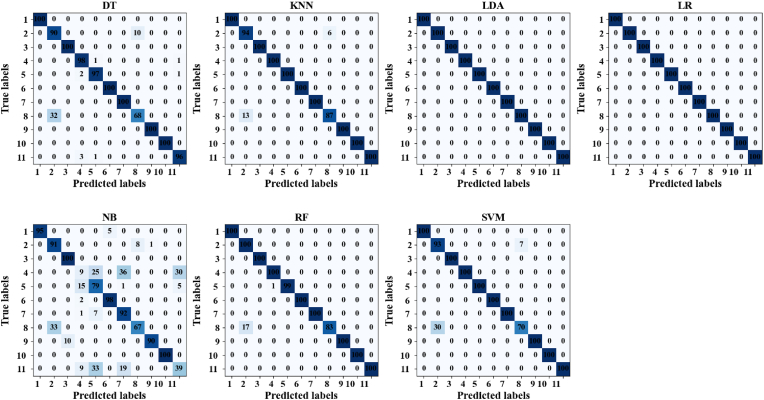
Confusion matric of the proposed model.

A confusion matrix is a performance evaluation tool used in classification tasks to visualize the performance of a machine learning model are shown in Fig. 8. It provides a comprehensive summary of the model's predictions by comparing them with the actual ground truth labels across different classes. The matrix is square and has dimensions equal to the number of classes in the classification problem. Each row of the matrix represents the instances in a predicted class, while each column represents the instances in an actual class. The diagonal elements of the matrix represent the number of correctly classified instances for each class, while off-diagonal elements indicate misclassifications. By analyzing the confusion matrix, one can gain insights into the model's strengths and weaknesses, such as its ability to distinguish between different classes and the types of errors it makes. In the classification experiments conducted using various machine learning algorithms, the confusion matrices provide valuable insights into the performance of each model. Across all classifiers, a remarkable level of accuracy is evident, with most showing near-perfect classification across multiple classes. Notably, DT, LDA, LR, RF, and SVM demonstrate flawless classification accuracy, each achieving 100% correct predictions across all classes. KNN exhibits high overall accuracy, albeit with a slight misclassification in a subset of samples. However, Naive Bayes showcases more variability, with noticeable misclassifications observed, particularly in instances of overlapping features.

## 5 Comparison with state-of-the-art techniques

Table 4 provides an extensive overview of different approaches and how well they work to identify different kinds of oil adulteration. These methods range from conventional analytical methods to sophisticated spectroscopic and imaging methods. Every technique has different accuracy levels and uses different technologies for analysis. The identification, categorization, and adulteration of camellia oil were examined by Wang et al. (2019). NIR methods in conjunction with chemometrics were employed by Gurtz et al. (Gertz et al., 2020) as a reliable analytical and statistical tool for evaluating virgin olive oil (VOO). In order to investigate sugar adulteration, Phillips et al. (Phillips and Abdulla, 2023) developed a unique method for honey fraud detection using hyperspectral imaging and machine learning. Among the notable additions is a brand-new feature smoothing method designed specifically for classification. In order to quantify the amount of starch present in adulterated fresh cheese, Barreto et al. (2018) proposed a study that used Hyperspectral Imaging (HSI) and partial least squares regression (PLSR) methodology to model the starch content. The integrity of vegetable oil was discussed by Wu et al. (2023), particularly in areas where concerns about authenticity are raised by economic considerations. They carried out a qualitative and quantitative investigation of adulteration in different vegetable oils using 3D fluorescence spectroscopy. In their study, Tachie et al. (2024) assessed how well machine learning (ML) techniques combined with Attenuated Total Reflectance Fourier Transform Infrared (ATR-FTIR) spectroscopy could distinguish and classify different oils and margarines, including those that were adulterated with canola-flaxseed oil and sunflower oil.

The adulteration of Camellia oil (CAO) was discussed by Chen et al. (2023), who concentrated on creating intelligent analysis methods for determining the categories and amounts of adulterated oil in CAO. Visible/Near Infrared Spectroscopy (Vis/NIRS) in conjunction with multivariate chemometrics was employed by Feng et al. (Srata et al., 2019) to assess superior engine oil samples that had been tampered with. Kaufmann et al. (2022) focused on verifying the authenticity of coriander oil and identifying adulteration with palm olein, canola oil, and soybean oil. PCA effectively distinguished between pure oils, while LDA and KNN algorithms were used for classification. Partial Least Squares (PLS) regression models demonstrated strong coefficients of determination (R2) ranging from 0.98 to 0.99 for detecting adulterated coriander oils, indicating suitability for quality control during processing. Du et al. (2021) introduces a novel real-time method for quantitatively detecting adulteration in coriander essential oil (CAO) using in situ near-infrared (NIR) spectroscopy and chemometrics. Discriminant analysis (DA) achieved a 96.7% accuracy in identifying adulterated oils, while partial least squares (PLS) regression, optimized with various pretreatment methods, demonstrated excellent predictive performance for adulteration levels, with determination coefficients (R2) exceeding 0.995 and low root mean square errors of calibration (RMSEC) and prediction (RMSEP). Vieira et al. (2021) were proposed a portable NIR spectrometer was utilized to verify the authenticity of pure sacha inchi oil. One-class models were employed for the untargeted detection of sacha inchi oil adulteration. DD-SIMCA and OC-PLS models achieved sensitivities exceeding 87%.

## 6 Conclusions

This paper aims to evaluate the feasibility of identifying the pure and adulterated oil using HSI with a spectral range of 400–1000 nm. HSI series of 670 oil samples were acquired under both UV and halogen illumination settings. HSI has emerged as a promising technique for oil adulteration detection and classification. By leveraging the unique spectral signature of different oil types and their mixtures, HSI provides a non-destructive and rapid means of distinguishing pure oils from adulterated ones. The ability to detect adulteration at various stages of the supply chain can have significant implications for ensuring the authenticity and quality of oils, safeguarding consumer's health, and protecting the reputation of producers. Machine learning is a powerful technique for oil adulteration detection and classification that combines multiple base classification (SVM, LR, RF, DT, LDA, ANN, KNN and Naïve Bayes) models to make more accurate and robust predictions. With further refinements and validation, hyperspectral imaging-based adulteration detection could become a key component in the battle against food fraud and adulteration in the coming years.

## CRediT authorship contribution statement

**Muhammad Aqeel:** contributed to the, Conceptualization, Data curation, Software, development, Methodology, Writing – original draft, preparation, and, Validation, of the study. **Ahmad Sohaib:** contributed to the, Data curation, Formal analysis, and, Visualization, of the study. **Muhammad Iqbal:** contributed to the, Investigation, Supervision, Validation, Software, development, and, Resources, of the study. **Hafeez Ur Rehman:** contributed to the, Investigation, Validation, review, and editing of the manuscript. **Furqan Rustam:** contributed to the, Project administration, and, Funding acquisition, review, and editing of the manuscript of the study.

## Declaration of competing interest

The authors declare that they have no known competing financial interests or personal relationships that could have appeared to influence the work reported in this paper.

## Data Availability

Data will be made available on request.

## References

[bib1] Abed H.M., Khairy H.L. (2023). The effect of adding pumpkin seed oil on physicochemical and sensory properties of the mayonnaise. IOP Conf. Ser. Earth Environ. Sci..

[bib2] Abed H.M., Luma Khairy H. (2023). The effect of adding pumpkin seed oil on physicochemical and sensory properties of the mayonnaise. IOP Conf. Ser. Earth Environ. Sci..

[bib3] Barreto A., Cruz-Tirado J.P., Siche R., Quevedo R. (2018). Determination of starch content in adulterated fresh cheese using hyperspectral imaging. Food Biosci..

[bib4] Basri K.N., Hussain M.N., Bakar J., Sharif Z., Khir M.F.A., Zoolfakar A.S. (2017). Classification and quantification of palm oil adulteration via portable NIR spectroscopy. Spectrochim. Acta Part A Mol Biomol Spectrosc.

[bib5] Chaudhari B.A., Patel M.P., Dharajiya D.T., Patel A.M., Thakur M.R. (2021).

[bib6] Chen A.-Q., Wu H.-L., Wang T., Wang X.-Z., Sun H.-B., Yu R.-Q. (2023). Intelligent analysis of excitation-emission matrix fluorescence fingerprint to identify and quantify adulteration in camellia oil based on machine learning. Talanta.

[bib7] Choudhary A., Gupta N., Hameed F., Choton S. (2020). An overview of food adulteration: concept, sources, impact, challenges and detection. Int. J. Chem. Stud..

[bib8] Cruz-Tirado J.P., Muñoz-Pastor D., de Moraes I.A., Lima A.F., Godoy H.T., Barbin D.F. (2023). Comparing data driven soft independent class analogy (DD-SIMCA) and one class partial least square (OC-PLS) to authenticate sacha inchi (Plukenetia volubilis L.) oil using portable NIR spectrometer. Chemometr. Intell. Lab. Syst..

[bib9] da Cruz Souza J., Soares S.F.C., de Paula L.C.M., Coelho C.J., de Araújo M.C.U., da Silva E.C. (2023). Bat algorithm for variable selection in multivariate classification modeling using linear discriminant analysis. Microchem. J..

[bib10] Du Q., Zhu M., Shi T., Luo X., Gan B., Tang L. (2021). Adulteration detection of corn oil, rapeseed oil and sunflower oil in camellia oil by in situ diffuse reflectance near-infrared spectroscopy and chemometrics. Food Control.

[bib11] Gertz C., Matthäus B., Willenberg I. (2020). Detection of soft‐deodorized olive oil and refined vegetable oils in virgin olive oil using near infrared spectroscopy and traditional analytical parameters. Eur. J. Lipid Sci. Technol..

[bib12] Gonzalez-Ortega R., Rajagukguk Y.V., Ferrentino G., Morozova K., Scampicchio M. (2024). Detection of butter adulteration with palm stearin and coconut oil by differential scanning calorimetry coupled with chemometric data analysis. Food Control.

[bib13] Heuscher S.A., Brandt C.C., Jardine P.M. (2005). Using soil physical and chemical properties to estimate bulk density. Soil Sci. Soc. Am. J..

[bib14] Johnson R. (2014).

[bib15] Kaufmann K.C., Sampaio K.A., García-Martín J.F., Barbin D.F. (2022). Identification of coriander oil adulteration using a portable NIR spectrometer. Food Control.

[bib16] Lima R. da S., Block J.M. (2019). Coconut oil: what do we really know about it so far?. Food Qual Saf.

[bib17] Lozano-Garzón K., Orduz-Díaz L.L., Guerrero-Perilla C., Quintero-Mendoza W., Carrillo M.P., Cardona-Jaramillo J.E.C. (2023). Comprehensive characterization of oils and fats of six species from the Colombian amazon region with industrial potential. Biomolecules.

[bib18] Lu C.-H., Li B.-Q., Jing Q., Pei D., Huang X.-Y. (2023). A classification and identification model of extra virgin olive oil adulterated with other edible oils based on pigment compositions and support vector machine. Food Chem..

[bib19] Mohammadinia F., Ranjbar A., Kafi M., Keshavarz R. (2023). Application of machine learning algorithms in classification the flow units of the Kazhdumi reservoir in one of the oil fields in southwest of Iran. J. Pet. Explor. Prod. Technol..

[bib20] Pal D. (2011).

[bib21] Phillips T., Abdulla W. (2023). A new honey adulteration detection approach using hyperspectral imaging and machine learning. Eur. Food Res. Technol..

[bib22] Portarena S., Leonardi L., Scartazza A., Lauteri M., Baldacchini C., Farinelli D. (2019). Combining analysis of fatty acid composition and δ13C in extra-virgin olive oils as affected by harvest period and cultivar: possible use in traceability studies. Food Control.

[bib23] Posada De La Paz M., Philen R.M., Borda I.A. (2001). Toxic oil syndrome: the perspective after 20 years. Epidemiol. Rev..

[bib24] Raj R.A., Sarathkumar D., Venkatachary S.K., Andrews L.J.B. (2023). 2023 3rd Int. Conf. Artif. Intell. Signal Process..

[bib25] Review A.C. (2023).

[bib26] Saifullah S., Prasetyo D.B., Dreżewski R., Dwiyanto F.A. (2023). Palm oil maturity classification using K-nearest neighbors based on RGB and L* a* b color extraction. Procedia Comput. Sci..

[bib27] Samuel D.S., Geetha R.V. (2014). Antioxidant activity of dry fruits: a short review. Res. J. Pharm. Technol..

[bib28] Srata L., Farres S., Fethi F. (2019). Engine oil authentication using near infrared spectroscopy and chemometrics methods. Vib. Spectrosc..

[bib29] Tachie C.Y.E., Obiri-Ananey D., Alfaro-Cordoba M., Tawiah N.A., Aryee A.N.A. (2024). Classification of oils and margarines by FTIR spectroscopy in tandem with machine learning. Food Chem..

[bib30] Tie Y., Rui X., Shi-Hui S., Zhao-Kai H., Jin-Yu F. (2024). A real-time intelligent lithology identification method based on a dynamic felling strategy weighted random forest algorithm. Petrol. Sci..

[bib31] Vieira L.S., Assis C., de Queiroz M.E.L.R., Neves A.A., de Oliveira A.F. (2021). Building robust models for identification of adulteration in olive oil using FT-NIR, PLS-DA and variable selection. Food Chem..

[bib32] Wahrburg U., Kratz M., Cullen P. (2002). Mediterranean diet, olive oil and health. Eur. J. Lipid Sci. Technol..

[bib33] Wang T., Wu H.-L., Long W.-J., Hu Y., Cheng L., Chen A.-Q. (2019). Rapid identification and quantification of cheaper vegetable oil adulteration in camellia oil by using excitation-emission matrix fluorescence spectroscopy combined with chemometrics. Food Chem..

[bib34] Wang B., Sun J., Xia L., Liu J., Wang Z., Li P. (2023). The applications of hyperspectral imaging technology for agricultural products quality analysis: a review. Food Rev. Int..

[bib35] Wei J.-W., He J.-R., Chen S.-Y., Guo Y.-H., Huo X.-Z., Zheng N. (2023). Synchronous fluorescence spectra-based machine learning algorithm with quick and easy accessibility for simultaneous quantification of polycyclic aromatic hydrocarbons in edible oils. Food Control.

[bib36] Wu M., Li M., Fan B., Sun Y., Tong L., Wang F. (2023). A rapid and low-cost method for detection of nine kinds of vegetable oil adulteration based on 3-D fluorescence spectroscopy. Lebensm. Wiss. Technol..

